# Development and validation of an integrative 54 biomarker-based risk identification model for multi-cancer in 42,666 individuals: a population-based prospective study to guide advanced screening strategies

**DOI:** 10.1186/s40364-025-00812-z

**Published:** 2025-08-11

**Authors:** Renjia Zhao, Huangbo Yuan, Yanfeng Jiang, Zhenqiu Liu, Ruilin Chen, Shuo Wang, Linyao Lu, Ziyu Yuan, Zhixi Su, Qiye He, Kelin Xu, Tiejun Zhang, Li Jin, Ming Lu, Weimin Ye, Rui Liu, Chen Suo, Xingdong Chen

**Affiliations:** 1 State Key Laboratory of Genetics and Development of Complex Phenotypes, ZhangjiangFudan International Innovation Center, Human Phenome Institute, Fudan University, Songhu Road 2005, Shanghai, 200433 China; 2 Fudan University Taizhou Institute of Health Sciences, Taizhou, China; 3 Department of Epidemiology, School of Public Health, Fudan University, Shanghai, China; 4 Singlera Genomics (Shanghai) Ltd, Shanghai, 200032 China; 5 Department of Biostatistics, School of Public Health, Fudan University, Shanghai, China; 6 Clinical Epidemiology Unit, Qilu Hospital of Shandong University, Jinan, Shandong, 250012 China; 7 Department of Medical Epidemiology and Biostatistics, Karolinska Institute, Stockholm, Sweden; 8 National Clinical Research Center for Aging and Medicine, Huashan Hospital, Fudan University, Shanghai, China; 9 Yiwu Research Institute of Fudan University, Yiwu, China

**Keywords:** Early cancer detection, Prospective follow-up study, Risk stratification model, Yield rate, Precision medicine, Cohort study

## Abstract

**Background:**

Early identification of high-risk individuals is crucial for optimizing cancer screening, particularly when considering expensive and invasive methods such as multi-omics technologies and endoscopic procedures. However, developing a robust, practical multi-cancer risk prediction model that integrates diverse, multi-scale data and with proper validation remains a significant challenge.

**Methods:**

We initialized the FuSion study by recruiting 42,666 participants from Taizhou, China, with a discovery cohort (*n* = 16,340) and an independent validation cohort (*n* = 26,308) after exclusion criteria. We integrated multi-scale data from 54 blood-derived biomarkers and 26 epidemiological exposures to develop a risk prediction model for five common cancers, including lung, esophageal, liver, gastric, and colorectal cancer. Employing five supervised machine learning approaches, we used a LASSO-based feature selection strategy to identify the most informative predictors. The model was trained and internally validated in the discovery cohort, externally applied in the validation cohort, and further evaluated through a prospective clinical follow-up to assess cancer events via clinical examinations.

**Results:**

The final model comprising four key biomarkers along with age, sex, and smoking intensity, achieving an AUROC of 0.767 (95% CI: 0.723–0.814) for five-year risk prediction. High-risk individuals (17.19% of the cohort) accounted for 50.42% of incident cancer cases, with a 15.19-fold increased risk compared to the low-risk group. During follow-up of 2,863 high-risk subjects, 9.64% were newly diagnosed with cancer or precancerous lesions. Notably, cancer detection in the high-risk group was 5.02 times higher than in the low-risk group and 1.74 times higher than in the intermediate-risk group. In particular, the incidence of esophageal cancers in the high-risk group was 16.84 times that of the low-risk group.

**Conclusions:**

This is the first population-based prospective study in a large Chinese cohort that leverage multi-scale data including biomarkers for multi-cancer risk prediction. Our effective risk stratification model not only enhances early cancer detection but also lays the foundation for the targeted application of advanced screening methods, including but not limited to multi-omics technologies and endoscopy. These findings support precision prevention strategies and the optimal allocation of healthcare resources.

**Supplementary Information:**

The online version contains supplementary material available at 10.1186/s40364-025-00812-z.

## Introduction

Cancer remains a major public health challenge, particularly in low- and middle-income countries [1]. According to GLOBOCAN 2020 statistics [2], half of all cancer cases and 58.3% of cancer-related deaths were estimated to occur in Asia, with China accounting for 4.56 million incidences and 3.00 million deaths. Despite advancements in cancer treatment, the prognosis of many malignancies remains poor due to delayed detection [3]. Early identification of high-risk individuals is essential for improving survival rates and optimizing healthcare resource allocation.

Current cancer screening strategies predominantly rely on single-cancer risk models that assess specific biomarkers [4–7]. While effective for targeted screening, these approaches often require multiple independent tests, leading to increased costs and logistical challenges in large-scale implementation. Established clinical screening modalities, such as low-dose computed tomography (LDCT) for lung cancer and endoscopic procedures for gastrointestinal malignancies, offer limited sensitivity in early-stage disease detection and are often underutilized due to their invasiveness and high cost. Furthermore, emerging multi-cancer detection models based on epigenomics [8–11] and proteomics [12] have shown promise in early cancer identification. However, their widespread adoption is hindered by high costs and the need for further validation in large prospective cohorts.

To address these challenges, we developed a population-based multi-cancer risk stratification model leveraging routinely collected epidemiological data and blood biomarkers. Using data from the FuSion (the integrative study by **Fu**dan and **Si**nglera for cancer early detecti**on**) project, a large-scale prospective study in China, we integrated machine learning-based variable selection to construct a cost-effective predictive model for five major cancers: lung, esophageal, gastric, liver, and colorectal cancer. Our study aims to provide a practical tool for early cancer risk assessment, facilitating targeted prevention strategies and improving screening efficiency in high-risk populations.

In this study, we collected 80 medical indicators, including 26 epidemiological questionnaire-based exposures and physical examination results, and 54 blood biomarkers. Various supervised machine learning methods were employed for variable selection and model construction. We developed and validated a multi-cancer risk prediction model in a discovery cohort, which was subsequently applied to a validation cohort to stratify individuals into different risk groups based on their 5-year incidence probability. Additionally, a prospective follow-up was conducted among at-risk participants, involving clinical medical examinations such as LDCT, gastroscopy, and abdominal ultrasonography. This approach allowed us to assess the model’s performance in real-world settings and evaluate its cancer yield rates, enabling the early detection of cancers or precancerous lesions.

## Methods

### Study participants and variable collection

The FuSion cohort is a population-based prospective study derived from the Taizhou Longitudinal Study (TZL) [13, 14], a community-based cohort initiated in 2007 in Taizhou, China. Participants aged 40 to 75 years were recruited in two phases: 16,340 individuals from September 2011 to January 2014 (the discovery cohort) and 26,308 individuals from July 2018 to November 2021 (the validation cohort). Baseline data collection included face-to-face interviews, physical examinations, and blood sampling. In addition, peripheral blood samples (8 to 10 mL) were collected in K2 EDTA vacutainers and stored at 4 °C until processing at the end of the day. After centrifugation, the plasma was separated and aliquoted into barcoded cryovials. The samples were then stored at − 80 °C or lower. Biochemical measurements were conducted between June and September 2024 in Shanghai Fourth People's Hospital, China.

We collected 26 epidemiological questionnaire-based exposures and physical examination results, and 54 blood biomarkers. All variables covering five major categories: (1) demographic characteristics (e.g., age, sex); (2) lifestyle and behavioral factors (e.g., smoking, alcohol consumption); (3) physical measurements (e.g., height, weight); and (4) biochemical and immunological markers (e.g. blood count, cancer biomarkers) discerned from blood test. The blood biomarkers includes albumin (ALB), albumin globulin ratio (A/G), alkaline phosphatase (ALP), alanine transaminase (ALT), alpha-fetoprotein (AFP), anti-H. pylori (Anti-HP), anti-hepatitis C virus antibody (HCV-Ab), aspartate carbamoyl transferase (AST), calcium (Ca), carbohydrate antigen 15–3 (CA-153), carbohydrate antigen 19–9 (CA-199), cancer antigen 125 (CA-125), carbon dioxide (CO2), carcinoembryonic antigen (CEA), chlorine (CL), amylase (AMY), creatinine (CREA), C-reactive protein (CRP), cystatin C (CYS-C), cytokeratin-19-fragment (CYFRA-211), direct bilirubin (DBIL), estimated glomerular filtration rate (EGFR), ferritin (FER), free T3 ELISA (FT3), free T4 ELISA (FT4), gamma-glutamyl transferase (GGT), glucose (GLU), globulin (GLOB), hepatitis B surface antigen (HBsAg), high-density lipoprotein (HDL), homocysteine (HCY), indirect bilirubin (IBIL), insulin (INS), kalium (K), low density lipoprotein (LDL), magnesium (Mg), neuron-specific enolase (NSE), pepsinogen I (PGI), pepsinogen I and pepsinogen II ratio (PGI/II), pepsinogen II (PGII), phosphorus (P), pro-gastrin-releasing peptide (ProGRP), sodium (Na), squamous cell carcinoma related antigen (SCC-Ag), tetraiodothyronine (T4), thyroid-stimulating hormone (TSH), total bilirubin (TBIL), total cholesterol (TCHO), total protein (TP), triglyceride (TG), triiodothyronine (T3), urea (UREA), and uric acid (URAC).A detailed summary of these biomarkers is provided in Table S1. These biomarkers were selected based on a combination of previous literature review and expert recommendations from clinical oncologists at the collaborating hospital. Tumor-related markers with reported diagnostic or prognostic value were prioritized, while other routinely measured biomarkers were included based on clinical relevance and laboratory availability.

### Data preprocessing

Variables with > 20% missing values were excluded, and for pairs of highly correlated variables (correlation coefficient > 0.8), we removed the variable with the higher missing rate. Finally, 20 epidemiological exposures and 49 biomarkers were included (Epidemiological exposures: three oral health conditions, and three sleep condition and mental health exposures were excluded; biomarkers: IBIL, DBIL, TCHO, AST, TP were excluded). Definition of these epidemiological exposures were shown on Table S2, and the correlation heatmap among biomarkers were shown on Figure S1.

Then, missing values for categorical variables were imputed using the most frequent category, while for continuous variables with missing values, we employed the K-nearest neighbors (KNN) algorithm [15]. This method locates the 50 closest individuals based on Euclidean distances and uses their median values for imputation purposes. To ensure robustness, we further excluded values below the 0.1st percentile and above the 99.9th percentile for each blood biomarker. In downstream analyses, all biomarkers measured as continuous variables were standardized using Z-score transformation, with a mean of 0 and a standard deviation (SD) of 1, for the purpose of better conduct model fitting.

### Outcome

Our study focuses on main 5 types of cancer (5CAs) of the respiratory and digestive systems. Cancer outcomes were defined using ICD-10 codes and obtained from the Taizhou CDC and local medical security system. Diagnoses were confirmed through pathology reports, imaging findings, or clinical evaluations where applicable. The 5CAs included lung cancer (ICD-10: C33/C34), esophageal cancer (ICD-10: C15), gastric cancer (ICD-10: C16), liver cancer (ICD-10: C22), and colorectal cancer (ICD-10: C18/C19/C20). These five cancers are the most prevalent non-sex-specific types in both China and Taizhou, accounting for more than 75% of all cancer cases annually [16]. Diagnoses of 5CAs are perceived as a single outcome. If multiple cancers were diagnosed in one participant, only the first recorded malignancy was considered. Only those who occurred cancer at least six months post-enrollment were considered valid incident cases. Follow-up duration was defined as the period from baseline enrollment until cancer diagnosis, study withdrawal, death, or the administrative censoring date of December 31, 2021.

### Prospective face-face follow-up

We conducted follow up of the population in the validation cohort from September 2022 to October 2023. Participants who voluntarily underwent medical examinations received LDCT scans [17, 18], gastroscopy, and abdominal ultrasonography [19, 20]. These procedures are clinically essential for diagnosing one of the 5CAs. If suspicious lesions are detected in the esophagus, stomach, or colorectum during endoscopic procedures, tissue samples will be taken for additional histopathological biopsy analysis. The cancer, precancerous lesion and suspicious pathological changes were diagnosed by clinical doctor, we carefully queried each participant undergoing examination, considering only those cancer diagnoses identified for the first time during this follow-up as valid. We also calculated yield rates for each type of cancers among groups.

### Statistical analysis

Continuous and categorical variables are delineated as means (SD) and frequencies (percentages), correspondingly. F-tests, χ2 tests, and Fisher’s exact tests were appropriately deployed to contrast differences between participants exhibiting cancer incident and those devoid. For the discovery cohort, individuals would be randomly divided into training and testing sets according to a 7:3 ratio. The training set is used for construction of the model, while the testing set is implemented to evaluate the predictive efficiency of the model, modify model parameters, and select the optimal model.

Our Penta-cancer Risk Identification Model (PRIME) was established as follows: Multivariable Cox proportional hazards regression models were employed to predictor selection and construct models. The best model was evaluated by the optimal Akaike Information Criterion (AIC) and number of variables included. We adopted stepwise regression and four machine learning procedures [21, 22] to select the optimal model. For stepwise regression, we applied a bidirectional selection procedure, beginning with forward selection followed by backward elimination, model selection was guided by the AIC, where lower AIC values indicate better model fit while balancing model complexity. Additionally, four supervised machine learning procedures were applied to fit the data and perform comparisons. These methods included L1 Absolute Shrinkage and Selection Operator (LASSO) regression, Partial Least Squares (PLS) regression, Random Forest (RF), and Support Vector Machine (SVM). LASSO method was harnessed for multivariable analysis with L1-penalized least absolute shrinkage and selection regression and was corroborated through tenfold cross-validation, it also utilizes a Cox regression model that penalizes the absolute magnitude of the coefficients in the regression model, with larger penalties effectuating the shrinkage of weaker factors towards zero and leaving only the most potent predictors within the model.

A 5-year incidence probability is established as the cancer prediction timeframe, with the risk probability threshold ascertained within the testing set. The 5-year relative risk score for an individual was calculated using the final multivariable Cox regression model, and the absolute 5-year cancer risk was estimated as follows:

$${Risk Score}_{i}=\sum_{j=1}^{n}{\beta }_{j}*{(x}_{ij}-{\overline{x} }_{j})$$

where Risk Score_i_ is the linear predictor for the i‑th individual; n is the number of variables included in model, ${\upbeta }_{\text{j}}$ is the Cox regression coefficient for the j‑th variable; ${\text{x}}_{\text{ij}}$ is the observed value of the j‑th variable for the i‑th individual; ${\overline{x} }_{j}$ is the mean of variable j in the training data.

And the absolute risk was calculated by:

$${Absolute Risk}_{i}(t=5)=1-\text{exp}(-{H}_{0}\left(5\right)*\text{exp}\left({Risk Score}_{i}\right))$$

where i means i‑th individual, H_0_(5) represents the cumulative baseline hazard function at time 5 years, and risk score means the individual linear predictor by the Cox model.

We will define low-risk group as lower than half of the median risk probability and those higher than three times of median was regarded as high-risk population. Conversely, the validation cohort will serve entirely as an independent validation set, facilitating the construction of incidence risk scores to subsequently discern high-risk individuals. The discrimination was evaluated using the area under the receiver operating characteristic curve (AUROC). To evaluate to what extend biomarkers could improve the predictive ability than epidemiological exposures, we use reclassification improvement (NRI), as well as the integrated discrimination improvement (IDI).

To better external promotion of our model, we developed a point score-based risk prediction system derived from the original PRIME model. The development of this scoring system followed established recommendations from previous literature [23–25]. In brief, categorical variables were grouped based on their inherent categories, while cut-off points for continuous variables were determined according to clinical relevance and classification standards provided by WHO sources. Model calibration was evaluated using the Hosmer–Lemeshow goodness-of-fit test, which assesses the agreement between observed and expected outcomes in subgroups of predicted risk. The observed number of events was then compared to the expected number within each group using a Chi-square statistic. A non-significant P-value suggests that the model fits the data well, with no significant deviation between observed and expected outcomes.

**Fig. 1:**
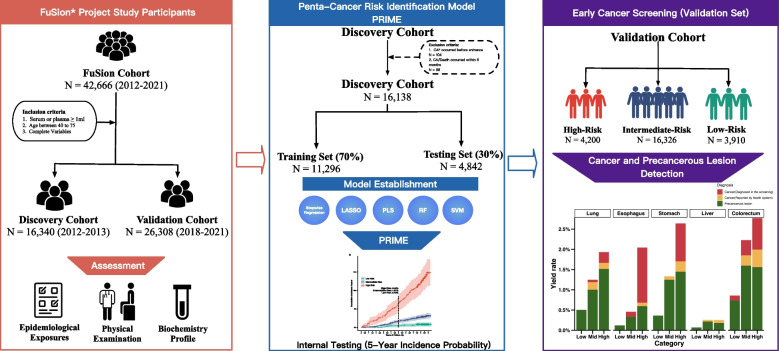
Study design and workflow. LASSO: L1 Absolute Shrinkage and Selection Operator (LASSO); PLS: Partial Least Squares; RF: Random Forest; SVM: Support Vector Machine. CA: cancer

In this study, two-sided P-value lower than 0.05 was regarded as statistical significance. All analysis were performed with R (V4.2.2). Survival analysis and prediction was calculated by the R package “survival”. Six sensitivity analyses were performed to confirm robustness. Figure 1 showed the flowchart of our study, after exclusion criteria, we finally adopted 16,138 and 26,058 participants in discovery cohort and validation cohort, respectively.

## Result

### Baseline characteristics

The discovery cohort had a median follow-up duration of 8.98 years, and 167 lung cancers, 132 esophageal cancers, 137 gastric cancers, 74 liver cancers, and 85 colorectal cancers were diagnosed among 16,138 participants. The crude and age-standardized incidence rates for each cancer type are presented in Table S3.

In the validation set, 163 out of 26,058 were diagnosed with cancer during a median follow-up of 2.01 years. Table 1 provides a detailed comparison of demographic and clinical characteristics between the discovery and validation cohorts. Participants in the validation cohort were generally older at baseline and had a higher proportion of males compared to those in the discovery cohort.

**Table 1:** Baseline characteristics of study population

Variables	Discovery cohort	Validation cohort
	*N* = 16,138	*N* = 26,058
**Follow-up Years (Median, IQR)**	8.98 (8.63–9.32)	2.01 (1.03–2.47)
**Age (mean (SD))**	53.06 (7.71)	55.27 (7.69)
**Age group (%)**		
−50	6268 (38.8)	6251 (24.0)
−60	5823 (36.1)	11,509 (44.2)
−70	3943 (24.4)	7921 (30.4)
70 +	104 (0.6)	377 (1.4)
**Sex (%)**		
Female	10,112 (62.7)	16,123 (61.9)
Male	6026 (37.3)	9935 (38.1)
**Marital status (%)**		
Married	15,039 (93.2)	23,992 (92.1)
Divorced/Widowed	981 (6.1)	1854 (7.1)
Single	118 (0.7)	205 (0.8)
Missingness	0(0.0)	7 (0.0)
**Education status (%)**		
Advanced	514 (3.2)	345 (1.3)
Intermediate	2020 (12.5)	2497 (9.6)
Basic	10,824 (67.1)	18,195 (69.8)
Less than basic	2780 (17.2)	5019 (19.3)
Missingness	0(0.0)	2 (0.0)
**Height (mean (SD)), cm**	159.48 (7.63)	159.72 (7.83)
**Weight (mean (SD)), kg**	62.72 (9.98)	63.57 (10.29)
**Tobacco smoking status (%)**		
Never	10,775 (66.8)	17,038 (65.4)
Ever	678 (4.2)	3709 (14.2)
Smoker	3834 (23.8)	5299 (20.3)
Missingness	851 (5.3)	12 (0.0)
**Smoking pack-years (mean (SD)), unit**	6.10 (12.46)	6.77 (13.78)
**Use filter when smoking (%)**		
No	1547 (9.6)	3246 (12.5)
Yes	2751 (17.0)	4146 (15.9)
Do not smoking/Missingness	11,840 (73.4)	18,666 (71.6)
**Inhalation smoking (%)**		
Oral cavity	1382 (8.6)	2877 (11.0)
Throat	821 (5.1)	1190 (4.6)
Lung	2254 (14.0)	3332 (12.8)
Do not smoking/Missingness	11,681 (72.4)	18,659 (71.6)
**Drinking status (%)**		
Never	12,362 (76.6)	18,108 (69.5)
Ever	345 (2.1)	2867 (11.0)
Drinker	3343 (20.7)	5071 (19.5)
Missingness	88 (0.5)	12 (0.0)
**Tea consumption status (%)**		
No	3516 (21.8)	4517 (17.3)
Yes	12,527 (77.6)	19,098 (73.3)
Missingness	95 (0.6)	2443 (9.4)
**Heavy activity works (%)**		
No	13,621 (84.4)	21,330 (81.9)
Yes	2370 (14.7)	4106 (15.8)
Missingness	147 (0.9)	622 (2.4)
**Moderate activity works (%)**		
No	4639 (28.7)	4067 (15.6)
Yes	11,353 (70.3)	21,713 (83.3)
Missingness	146 (0.9)	278 (1.1)
**Moderate walk every day (%)**		
No	6362 (39.4)	10,571 (40.6)
Yes	9135 (56.6)	15,016 (57.6)
Missingness	641 (4.0)	471 (1.8)
**Waist (mean (SD)), cm**	82.56 (8.86)	86.92 (9.17)
**Hip (mean (SD)), cm**	94.63 (6.07)	97.16 (6.13)
**Waist-Hip ratio (mean (SD)), unit**	0.87 (0.06)	0.89 (0.06)
**Body Fat (mean (SD)), percentage**	31.10 (5.71)	31.85 (5.53)
**Visceral fat (mean (SD)), percentage**	9.48 (4.29)	9.76 (4.39)
**Pulse (mean (SD)), times per minute**	77.93 (10.80)	75.51 (10.70)
**BMI (mean (SD)), unit**	24.60 (3.11)	24.86 (3.21)
**BMI category (%)**		
Underweight	202 (1.3)	322 (1.2)
Normal	6794 (42.1)	10,193 (39.1)
Overweight	6830 (42.3)	11,228 (43.1)
Obesity	2259 (14.0)	4298 (16.5)
Missingness	53 (0.3)	17 (0.1)
**Systolic blood pressure (mean (SD)), unit**	137.88 (19.54)	136.59 (19.10)
**Diastolic blood pressure (mean (SD)), unit**	109.17 (15.79)	84.49 (10.96)

^*^IQR Interquartile Range, *SD* Standard error

Prior to data processing, 49 biomarkers exhibited varying degrees of missingness, ranging from 0.01% (Cl) to 3.50% (PGI/II) (Figure S2). The summary of these biomarkers between groups was shown on Table S4. A forest plot illustrating the associations between each biomarker and cancer incidence is provided in Figure S3 and Table S5. A total of 30 biomarkers were found to be significantly associated with cancer incidence, including 23 risk factors and 7 protective factors, with CYFRA-211 showed the best statistical significance and highest predictive power (HR:1.32;95%CI:1.26–1.39, *P* < 2E-16).

### Development and performance of the PRIME model

Among the statistical and machine learning methods tested, LASSO Cox regression demonstrated superior model performance with an optimal balance between predictor inclusion and discrimination capability. (Delong: Z_Cox*vs.*LASSO_ = −0.87, *P* = 0.381; Z _RF*vs.*LASSO_ = −2.35, *P* = 0.02; Z_Cox*vs.*RF_ = 1.91, *P* = 0.05) (Figure S4 & Figure S5). Seven variables were retained in the final model: age (continues), sex, smoking pack-years (continues), AFP, CEA, CYFRA-211 and HBsAg (Table 2**).** In hence, the linear prediction risk score was calculated as follows:

**Table 2:** Penta-cancer Risk Prediction Model (PRIME) via LASSO Cox regression

Predictors	Coefficients	Hazard Ratio (95%CI)	Standard Error	Z-score	P-Value
Age (per 5 years)	0.422	1.53 (1.42–1.64)	0.038	11.083	< 0.001
Sex	0.558	1.75 (1.36–2.25)	0.128	4.372	< 0.001
Smoking pack-years	0.014	1.01 (1.01–1.02)	0.003	4.220	< 0.001
AFP	0.053	1.06 (1.03–1.08)	0.013	4.149	< 0.001
CEA	0.085	1.09 (1.06–1.12)	0.015	5.745	< 0.001
CYFRA-211	0.187	1.21 (1.12–1.30)	0.038	4.979	< 0.001
HBsAg	0.125	1.13 (1.04–1.23)	0.043	2.912	< 0.001

$${Risk Score}_{i}=0.422*{\text{Age }\left(\text{per }5\text{ years}\right)}_{i} +0.558*{\text{Sex}}_{i}+0.014*{\text{Smoking Pack}-\text{Years}}_{i} +0.053*{\text{AFP}}_{i}+0.085*{\text{CEA}}_{i}+0.187*{\text{CYFRA}-211}_{i}+0.125*{\text{HBsAg}}_{i}-4.565$$

In this formula, i means i-th individual, sex was coded as 1 for males and 0 for females, and all biomarkers were Z-score transformed value.

The H_0_(5) in the training set was 0.00932. The detailed parameter was provided in Table S6. Moreover, we have launched a web-based calculator that enables individuals to estimate their risk: https://fdu-renjiazhao.github.io/FuSion-Calculator/.

In brief, the most significant predictor was sex (HR male *vs.* female: 1.75; 95% CI = 1.36–2.25). Each five-year increase in age was associated with a 53% increased risk of cancer (HR = 1.53, 95% CI: 1.42–1.64). The NRI indicated that biomarkers significantly enhanced 29% of reclassification for the population as well as 24% improvements in non-incidents (*P* < 0.05), and IDI further proved that 9.2% of improvements for PRIME were found after adding biomarkers (*P* = 0.020) (Table 3).

**Table 3:** Discrimination of models for incident 5CAs in training set

Model	AUROC (95%CI)	NRI			IDI (%)	*P*-value
		**NRI (total)**	**NRI + **	**NRI-**		
Epidemiological model ^a^	0.752 (0.720–0.784)	/	/	/	0.4% (0.1%−0.9%)	** < 0.001**
Full model ^b^	0.789 (0.760–0.818)	29% (17%−38%)	5% (−7%−16%)	24% (11%−27%)	9.2% (0.8%−18.7%)	**0.020**

a: Predictors in the epidemiological model includes age, sex, and smoking pack-years

b: Predictors in the full model includes epidemiological model, plus AFP, CEA, CYFRA-211, and HBsAg

### Internal evaluation in the testing set

**Fig. 2:**
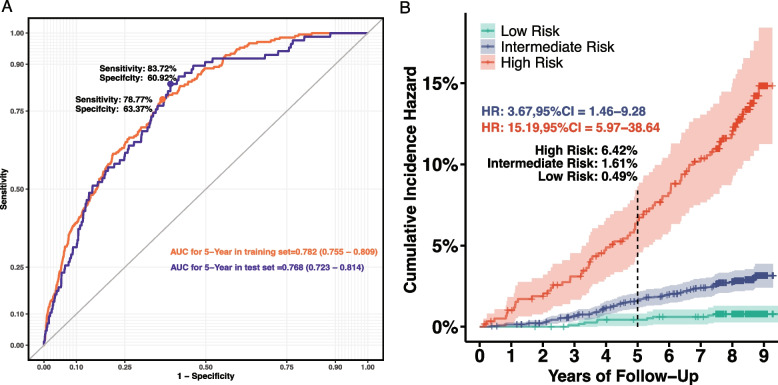
Five-year incidence probability prediction by PRIME. (**A**) AUROC for PRIME between training and testing sets. Orange line: ROC curve in training set; Dark blue line: ROC curve in testing set. (**B**) Cumulative survival curves. Red: High risk population; Blue: Intermediate risk population; Green: Low risk population. Black vertical dotted lines: Focus time to 5 years. AUROC: Area under the receiver operation curve; PRIME: Penta-cancer risk identification model

**Fig. 3:**
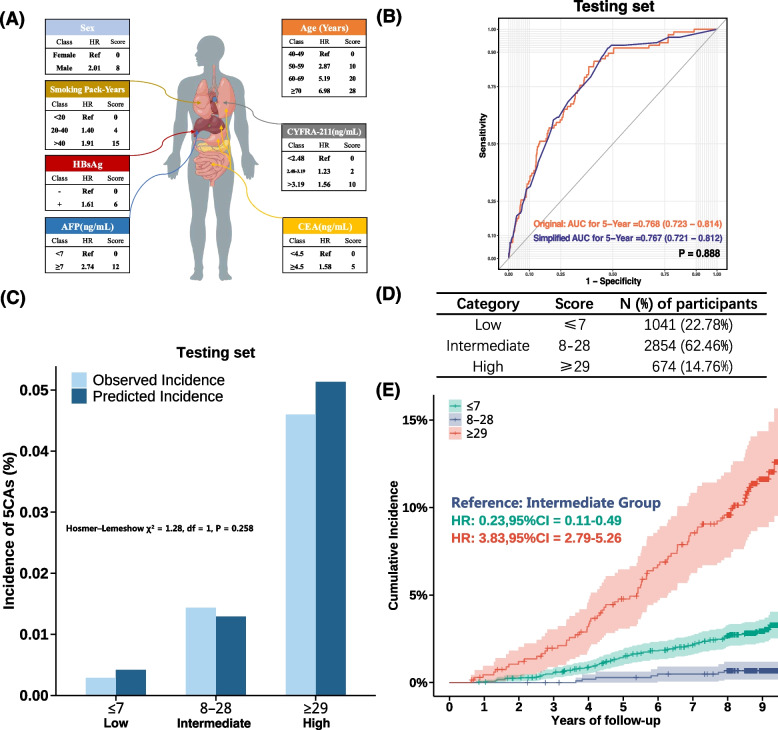
Simplified PRIME by point-based score. (**A**) The scoring system. (**B**) Model comparation between original PRIME and simplified PRIME for 5-Year probability in the testing set. (**C**) The calibration of the point-based risk score in the testing set. (**D**) Cut-off and proration of at-risk groups. (**E**) Cumulative hazard plot by point-based score PRIME. AFP: alpha fetoprotein; CYFRA-211: cytokeratin 19 fragment; CEA: carcinoembryonic antigen; HBsAg: hepatitis B surface antigen. Orange AUROC: Original PRIME’s AUROC in testing set. Blue AUROC: Simplified PRIME’s AUROC in testing set. Green Curve: Low risk group (Score ≤ 7); Blue Curve: Intermediate risk group (8 to 28); Red Curve: High risk group (≥ 29). The score was a sum of individual’s risk category via 7 predictors

A valid population of 4,569 individuals were retained within the testing set, with 86 incidents in 5 years. The PRIME model demonstrated robust discrimination in the testing set, with an AUROC of 0.768 (95% CI: 0.723–0.814; Sensitivity: 83.72%, Specificity: 60.92%, Fig. 2A). Ultimately, we determined two cut-off values: 0.646% and 3.878%. After a 5-year follow-up period, 43% of all cancer cases were expected to occur in the high-risk group, with a crude incidence rate approximately 16 times higher than in the low-risk group (Table 4). Cumulative incidence curves demonstrated a clear separation between risk groups, confirming the model’s stratification ability **(**Fig. 2B**).** Cox regression analysis indicated that individuals in the intermediate-risk group had a 3.67-fold increased risk (95% CI: 1.46–9.28, *P* < 0.05), while those in the high-risk group had a 15.19-fold increased risk (95% CI: 5.97–38.64, *P* < 0.001) compared to the low-risk population Fig. 3.

**Table 4:** Distribution, expected, and observed incident summary via 5-year incidence probability in FuSion testing set and validation set

Name	Low risk	Intermediate risk	High risk
**Testing set**			
*N* (%)	1163(25.45%)	2810(61.50%)	596(13.05%)
Observed incident (%)	5(5.81%)	44(51.16%)	37(43.03%)
Crude incidence rate (per 100,000)	429.92	1708.19	6879.19
Expected incident (%)	5(5.32%)	48(51.06%)	41(43.62%)
**Validation set**			
*N* (%)	3910(16.00%)	16,326(66.81%)	4200(17.19%)
Observed incident (%)	8(4.90%)	76(46.63%)	79(48.47%)
Expected incident after 5 years (%)	18(3.00%)	280(46.58%)	303(50.42%)
Enrichment ratio	Ref	3.73	15.67

To enhance the clinical applicability of the PRIME model, we developed a simplified risk assessment tool. The score was a sum of individual’s risk category via 7 predictors (Fig. 3A). In the testing set, the total score ranged from 0 to 61. The predictive performance of this streamlined version was statistically equivalent to that of the original model (Fig. 3B, *P* = 0.888). Moreover, the calibrations of the Cox model were acceptable as indicated by the Hosmer–Lemeshow statistics (Fig. 3C). As shown on Fig. 3D, based on the score distribution, we identified two new cut-off points at 7 and 29, categorizing participants into low-, intermediate-, and high-risk groups, accounting for 22.78%, 62.46%, and 14.76% of the population, respectively. The cumulative hazard across risk groups also showed a consistent pattern with the original model (Fig. 3E).

### External validation in the independent cohort

Applying the PRIME model to the validation cohort, 4,200 individuals (17.19%) were classified as high risk, while 16,326 (66.81%) and 3,910 (16.00%) were categorized as intermediate and low risk, respectively. The model estimated that 601 cancer cases would occur within five years, with 50.42% of all expected cases concentrated in the high-risk group. The cancer enrichment ratio for the high-risk group was 15.67-fold higher than that of the low-risk group (Table 4).

### Prospective follow-up

By the end of October 31, 2023, a total of 9,643 individuals participated in prospective follow-up screening, including 2,863 (68.17%) high-risk, 5,288 (32.39%) intermediate-risk, and 1,492 (38.15%) low-risk individuals. Compliance rates varied by screening modality, with 92.32% of participants undergoing LDCT for lung cancer screening and 95.73% undergoing abdominal ultrasonography for liver cancer detection, while only 45.08%–45.61% completed endoscopic evaluations.

**Fig. 4:**
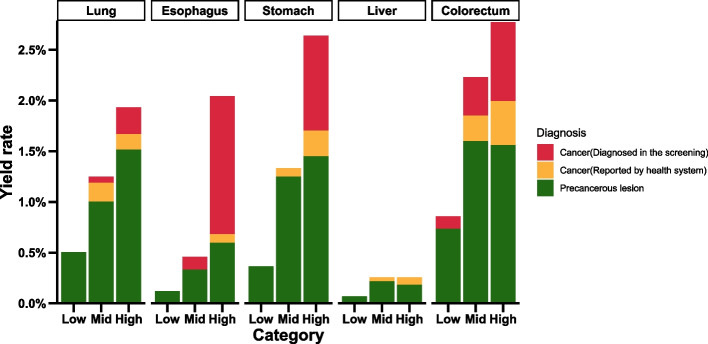
Summary of the yield rates for cancer and precancerous lesion at prospective follow-up. Red: newly diagnosed cancer incidence at follow-up; Yellow: cancer incidence provided by health system; Green: precancerous lesion

A total of 93 new cancer cases and 241 precancerous lesions were identified (Table S7). The cancer detection rate in the high-risk group was 9.64%, which was 5.02-fold higher than that in the low-risk group (1.92%). As shown on Fig. 4, among 5CAs, colorectal cancer had the highest detection rate (2.77%), with an enrichment ratio of 3.23-fold compared to the low-risk group. Esophageal cancer exhibited the most robust risk stratification performance, with a 2.04% detection rate in the high-risk group, corresponding to a 16.84-fold enrichment compared to the low-risk group. The high-risk group also exhibited an overall cancer enrichment capacity ranging from 1.12-fold (liver cancer) to 2.49-fold (esophageal cancer) compared to the general population.

For participants who completed the full five-cancer screening protocol (*N* = 4,137, Figure S6), the crude five-year incidence rate of the targeted cancers was 5,074.92 per 100,000 individuals, rising to 8,683.73 per 100,000 in the high-risk subgroup.

### Sensitivity analysis

Six sensitivity analyses were conducted to evaluate the robustness of the PRIME. First, to assess the impact of demographic exposures, a nested case–control study was performed by matching cases and controls in a 1:4 ratio based on five-year age groups and sex within the entire cohort. Ten significant predictors were identified, all of which were also selected in the PRIME model, meaning that biomarker effects were not covered up by demographic status (Table S8). Second, we excluded individuals who were diagnosed with one of the 5CAs within 2 and 3 years after baseline, respectively. The predictive performance remained robust in both scenarios (Table S9). Third, we applied the PRIME to each individual cancer type. All five cancer types demonstrated significant predictive power. Liver cancer showed the highest AUROC of 0.870 (95% CI: 0.807–0.933), while colorectal cancer had moderate performance (AUROC = 0.716, 95% CI: 0.610–0.822) (Table S10). Fourth, we performed stratified analyses by sex and age group. PRIME demonstrated better predictive performance in females than in males, and in younger participants compared to older ones (Table S11). Fifth, we plotted a curve illustrating the relationship between the number of high-risk individuals screened and the proportion of total cases captured. Our definition of the high-risk group falls within the steepest part of the curve, indicating a favorable balance between cancer detection yield and screening-related resource expenditure (Figure S7). Last, we compared the distribution between those “high-risk participants” who attend follow-up and those not, we found that those who attended had significant higher hazard characteristics in sex, smoking, AFP, and CEA, which may imply the selection bias (Table S12).

## Discussion

In this study, we utilized the FuSion cohort to develop and validate a multi-cancer risk stratification model, PRIME, that effectively predicts the occurrence of five major cancers. By identifying high-risk individuals within a general population, this model enables targeted cancer screening, thereby optimizing resource allocation and facilitating early cancer detection. Our results demonstrate that the PRIME model achieves robust discrimination, with high-risk individuals exhibiting up to a 15.19-fold increased cancer incidence compared to low-risk individuals. Prospective follow-up further validated its clinical utility, with the high-risk group showing a five-fold enrichment in cancer detection rates. By stratifying risk at the population level, PRIME provides a cost-effective, scalable, and clinically applicable tool for targeted cancer screening, optimizing healthcare resources while improving early detection outcomes.

While traditional cancer screening models primarily focus on individual cancer types and require multiple independent assessments. For instance, widely used models, such as PLCOm2012 for lung cancer, rely on smoking history and demographic variables, achieving AUROC values of approximately 80% in external validations [4, 26, 27]. However, these models remain limited to single-cancer risk prediction and often lack applicability to broader screening programs. Similarly, current pan-cancer detection strategies, primarily based on epigenomics and proteomics, demonstrate high specificity but remain costly and require further validation in large population-based cohorts. PRIME offers a unified, population-based risk stratification approach, with widely used machine learning AI-based model, which have been proved be more effective than traditional methods [28]. LASSO regression, as a machine learning–based variable selection method, performs both shrinkage and automatic elimination of less informative predictors. By penalizing the absolute size of regression coefficients, it effectively reduces model complexity and minimizes redundancy, thereby enhancing interpretability and generalizability of the final predictive model. This approach enhances its ability to identify high-risk individuals with greater accuracy while maintaining clinical interpretability. The model provides a simplified yet effective approach for multi-cancer risk prediction, enabling more efficient screening allocation.

It is worth noting the predictive ability of PRIME for each single cancer types. Specifically, PRIME demonstrated the highest discriminative ability for liver cancer. The final variables included via LASSO included HBsAg and AFP, both of which are clinically specific markers for liver injury and are widely used in hepatocellular carcinoma surveillance. This may partially explain the superior predictive performance observed for liver cancer. Nevertheless, despite slightly lower AUROC values for lung, esophageal, and gastric cancers compared to liver cancer, the model still demonstrated comparable or even superior predictive accuracy relative to previously published single-cancer models. This highlights the robustness and broad applicability of our multi-cancer prediction approach. Additionally, the model’s stratification power for colorectal cancer was relatively weaker compared to other type of cancers. Given that colorectal cancer risk is significantly influenced by dietary habits and gut microbiota, incorporating metabolomic and genetic risk factors could further refine future predictive accuracy [29–31]. Recently, several cancer early screening models based on ctDNA methylation have been proposed, with colorectal cancer being the primary target [32]. The results suggest that methylation models can achieve 80–99% predictive specificity for colorectal cancer, offering a validated approach for targeted screening in high-risk individuals [33, 34].

Our findings indicate that screening only 17% of the population identified as high-risk could detect over 50% of all incident cancer cases, a significant improvement in screening efficiency. However, defining individuals as “high-risk” must be cautious. Such labeling may induce psychological stress—an established risk factor for cancer—which could lead to indirect harmful health consequences [35]. During the follow-up, our staff actively communicate with participants, clearly explaining what “high-risk” means in statistical terms, shown to reduce anxiety and improve understanding.

Additionally, it is essential to balance screening efficacy with economic costs and minimize the other potential harms associated with over-screening. To address this, we conducted a sensitivity analysis and plotted an estimation curve illustrating the relationship between screening cost and detection efficiency. Based on this curve, researchers or policymakers can select appropriate risk score cutoffs tailored to their specific priorities—whether aiming for broader population coverage or a more cost-effective, targeted screening strategy.

The prospective follow-up design strengthens the level of evidence in our study, as the identification of incident cancer cases was diagnosed by the clinical gold standard. Notably, our validation cohort had a short-term follow-up with part of cancer diagnosis, which could help us evaluate the PRIME for long-term outcome (cancer). Combined with precancerous lesion as a short-term outcome. The result revealed that esophageal cancer showed the strongest risk stratification capability, with an enrichment ratio of 16.84-fold in the high-risk group compared to the low-risk group. However, it has to be noticed that only half of all participants attend follow-up agree to take endoscopy, and our sensitivity analysis showed that those who accepted screening had higher predicted risk probability than those not. We assume that it could introduce potential selection bias and overestimate the evaluation of true effect. Future studies with higher endoscopy accepted rates or population-based implementations are needed to validate the model’s performance. Overall, these findings underscore the potential of PRIME in enhancing precision prevention strategies, particularly for gastrointestinal malignancies.[36, 37].

This study has several key strengths. First, it represents the largest prospective, population-based study in China to develop and validate a multi-cancer risk prediction model. Unlike hospital-based case–control studies, this design reduces selection bias and enhances generalizability. Second strength of our study is the comprehensiveness of biomarker coverage, including a large panel of circulating proteins and metabolites, many of which are not currently measured in most of large-scale population cohorts. The rich molecular profiling offers a unique opportunity to explore multidimensional early-detection signals across multiple cancers. Moreover, the model underwent both internal and external validation, demonstrating robust performance across different subpopulations. Third, the prospective follow-up provided direct evidence of PRIME’s real-world applicability, confirming its ability to identify high-risk individuals who benefit from early cancer detection.

However, certain limitations should be acknowledged. First, biomarker stability over time could affected model performance [38, 39]. Blood samples for the discovery cohort were collected over seven years before biomarker measurements, potentially leading to biological degradation despite standard storage protocols. To address this, reliability testing of key biomarkers was conducted, demonstrating high correlation coefficients (70–94%), supporting the robustness of PRIME. Second, the study population was recruited from a single geographic region, potentially limiting its generalizability to other populations. Although the two cohorts cover different time periods, with baseline biological sample collection and storage conducted in different years, leading to potential batch and temporal variability. And there is a clear urban–rural composition difference between the two populations, representing distinct demographic and environmental exposures. These differences make the validation more meaningful than a random split within a single homogeneous cohort. For existing large-scale external cohorts, the depth of the biomarker panel varied from us, like UK Biobank, about 57% of them are not currently available. In all, more multi-center-based further validation across diverse ethnic and regional groups is necessary.

## Conclusion

The PRIME model provides a novel, cost-effective, and clinically feasible approach for multi-cancer risk stratification in a large population-based cohort. By leveraging routine epidemiological and blood biomarker data, PRIME enables the identification of high-risk individuals, facilitating targeted screening and early cancer detection. Prospective validation confirmed its clinical utility, demonstrating a five-fold enrichment in cancer detection rates among high-risk individuals.

## Supplementary Information

Supplementary material 1.

## Data Availability

The datasets used and analyzed during the current study are available from the corresponding author on reasonable request.

## Abbreviations

AFP: Alpha fetoprotein
AIC: Akaike information criterion
ALB: Albumin
ALP: Alkaline phosphatase
ALT: Alanine transaminase
AMY: Amylase
Anti-HP: Anti-H. pylori
AST: Aspartate carbamoyl transferase
AUROC: Area under the receiver operating characteristic curve
CA-125: Cancer antigen 125
CA-153: Carbohydrate antigen 15–3
CA-199: Carbohydrate antigen 19–9
CEA: Carcinoembryonic antigen
Cl: Chlorine
CREA: Creatinine
CRP: C-reactive protein
CYS-C: Cystatin C
CYFRA-211: Cytokeratin 19 fragment
DBIL: Direct bilirubin
EGFR: Estimated glomerular filtration rate
FER: Ferritin
FT3: Free T3 ELISA
FT4: Free T4 ELISA
FuSion: The integrative study by Fudan and Singlera for cancer early detection
GGT: Gamma-glutamyl transferase
GLOB: Globulin
GLU: Glucose
HbSAg: Hepatitis B surface antigen
HCV-Ab: Anti-hepatitis C virus antibody
HDL: High-density lipoprotein
HCY: Homocysteine
IBIL: Indirect bilirubin
INS: Insulin
K: Kalium
KNN: K-nearest neighbors
LASSO: L1 absolute shrinkage and selection operation
LDL: Low-density lipoprotein
LDCT: Low-dose computed tomography
Mg: Magnesium
Na: Sodium
NSE: Neuron-specific enolase
P: Phosphorus
PGI: Pepsinogen I
PGI/II: Pepsinogen I and pepsinogen II ratio
PGII: Pepsinogen II
PLS: Partial least squares
PRIME: Penta-cancer risk identification model
ProGRP: Pro-gastrin-releasing peptide
RF: Random forest
SCC-Ag: Squamous cell carcinoma related antigen
SVM: Support vector machine
T3: Triiodothyronine
T4: Tetraiodothyronine
TBIL: Total bilirubin
TCHO: Total cholesterol
TG: Triglyceride
TP: Total protein
TSH: Thyroid-stimulating hormone
TZL: Taizhou Longitudinal Study
URAC: Uric acid
UREA: Urea
