# Supplementary material for: Development and validation of an integrative 54 biomarker-based risk identification model for multi-cancer in 42,666 individuals: a population-based prospective study to guide advanced screening strategies
Source: Biomark Res. 2025 Aug 11;13:101. doi: 10.1186/s40364-025-00812-z (PMC12341305; doi:10.1186/s40364-025-00812-z)
Supplement: Supplementary file 1 — Supplementary material 1. [file 40364_2025_812_MOESM1_ESM.pdf]

Supplementary Materials For

Development and Validation of an Integrative 54 Biomarker-based Risk Identification Model for Multi-Cancer in 42,666 Individuals: A Population-Based Prospective Study to Guide Advanced Screening Strategies

Zhao et.al

Table of Contents

Table S1. Full name, abbreviation and normal reference range for all biomarkers 3

Table S2. Definition of epidemiological exposures in FuSion study 5

Table S3. Incident events and incidence rate of five common cancers in FuSion discovery cohort 9

Table S4. Summary of 49 biomarkers in discovery and validation cohort................................. 10

Table S5. Univariable Cox regression results for incident 5CAs... 12

Table S6. Variable definitions and transformation parameters for the PRIME model 15

Table S7. Definition and distribution of new diagnosis cancer or precancerous lesion of different type of cancer.. 16

Table S8. Significant variables in 1:4 matched subgroup 17

Table S9. Predictive ability of PRIME after restricted population after enrollment.................. 18

Table S10. Predictive ability of PRIME in each cancer types 18

Table S11. Stratified analysis of PRIME by sex and age group 18

Table S12. Comparation of high-risk participant between follow-up attendance status 18

Figure S1. Correlation heatmap among 54 biomarkers. 19

Figure S2. Missing counts (percentage, exposure name) of 49 biomarkers................................ 20

Figure S3. Forest plot of all 49 biomarkers with cancer incident risk.. 21

Figure S4. Model Comparison among five different machine learning procedures. (LASSO). ... 22

Figure S5. Variable Selection via L1 Absolute Shrinkage and Selection Operator (LASSO)...... 23

Figure S6. The Venn chart of people participating in medical examinations.............................. 24

Figure S7. Screening curve between participants needed to screen and percentage of cancer incidents captured. **25**

Table S1. Full name, abbreviation and normal reference range for all biomarkers

Full Name	Abbreviations	Lower normal reference range	Higher normal reference range
Urea	UREA	3.1	8
Uric Acid	URAC	208	428
Thyroid-stimulating hormone	TSH	0.35	5.1
Total Protein	TP	65	85
Triglyceride	TG	≤1.7	/
Total cholesterol	TCHO	≤5.18	/
Total bilirubin	TBIL	<23	/
Tetraiodothyronine	T4	64.4	186.6
Triiodothyronine	T3	0.89	2.49
Squamous cell carcinoma related antigen	SCC-Ag	<2	/
Pro-gastrin-releasing peptide	ProGRP	<78.62	/
Pepsinogen II	PGII	/	/
Pepsinogen I and Pepsinogen II Ratio	PGI/II	>3	/
Pepsinogen I	PGI	>70	/
Phosphorus	P	0.85	1.51
Neuron-specific enolase	NSE	<16.5	/
Sodium	Na	137	147
Magnesium	Mg	0.75	1.02
Low density lipoprotein	LDL	≤4.11	/
High-density lipoprotein	HDL	1.16	1.42
Kalium	K	3.5	5.3
Insulin	INS	15.27	173.63
Indirect bilirubin	IBIL	/	/
Homocysteine	HCY	≤15	/
Anti-hepatitis C virus antibody	HCV-Ab	<1.00	/
Hepatitis B surface antigen	HBsAg	<0.08	/
Glucose	GLU	3.9	6.1
Globulin	GLOB	20	40
Gamma-glutamyltransferase	GGT	10	60
Free T4 ELISA	FT4	11.19	23.81
Free T3 ELISA	FT3	2.76	6.45
Folate	FOL	7	46.4
Ferritin	FER	>3.20	/

Estimated glomerular filtration rate	EGFR	90	120
Direct Bilirubin	DBIL	<5	/
Cystatin C	CYS-C	0.59	1.03
Cytokeratin-19-fragment	CYFRA-211	<4.2	/
C-Reactive Protein	CRP	0	6
Creatinine	CREA	57	97
Carbon dioxide	CO2	20	31
Chlorine	CL	99	110
Carcinoembryonic antigen	CEA	<4.5	/
Carbohydrate antigen 19-9	CA-199	<36	/
Carbohydrate antigen 15-3	CA-153	<23	/
Cancer antigen 125	CA-125	<35	/
Calcium	Ca	2.11	2.52
Aspartate carbamoyltransferase	AST	15	40
Anti-H. pylori	Anti-HP	/	/
Amylase	AMY	35	135
Alanine transaminase	ALT	9	50
Alkaline phosphatase	ALP	45	125
Albumin	ALB	40	55
Alpha-Fetoprotein	AFP	<7.0	/
Albumin Globulin Ratio	A/G	1.2	2.4

Table S2. Definition of epidemiological exposures in FuSion study

Variable	Definition	Allowable values
**Age (year)**	The length of time an individual survives from the date of birth in the Gregorian calendar to the date of calculation, with the unit of measurement being years	Continuous
**Age group**	Categorized age groups	1: -50<br>2: -60<br>3: -70<br>4: 70+
**Sex**	An individual's biological sex	1: Male<br>2: Female<br>9: Unspecified
**Marital status**	The current marital status of an individual	10: Unmarried<br>20: Married<br>21: First marriage<br>22: Remarriage<br>30: Widowed<br>40: Divorce<br>90: Unspecified

**Education grade**	highest level of education received by an individual	1: Graduate student<br>2: Undergraduate degree<br>3: Junior colleges and junior colleges<br>4: High School<br>5: Secondary vocational schools or secondary technical schools<br>6: Technical school<br>7: Junior High School<br>8: Primary School<br>9: Preschool children 10: Illiterate or semi-illiterate
**Height**	Automatic measurement, height	Continuous
**Weight**	Automatic measurement, weight	Continuous
**Tobacco smoking status**	an individual's past and present smoking situations	1: Never<br>2: Yes, I'm still drawing now<br>3: I have smoked before and have quit smoking (for at least one year or more).
**Smoking pack-years**	The cumulative years of smoking multiplied by the number of smoking packs	Continuous

**Use filter when smoking**	whether an individual uses a filter tip when smoking	1: Yes<br>2: No
**Inhalation smoking**	The depth of an individual's smoking	1: Oral cavity<br>2: Throat area<br>3: Lungs
**Drinking status**	Has the individual ever consumed alcohol at least once a week within six months	Yes, still drinking alcohol now<br>Yes, I have quit now<br>3: No
**Tea consumption status**	Has the individual ever drunk at least one cup of tea every day for six months	1: Yes<br>2: No<br>3: Previous tea drinking
**Heavy activity works every day**	Whether the individual has engaged in any specific heavy physical activity in the past 7 days	1: Yes<br>2: No
**Moderate activity works**	Whether the individual has engaged in any specific moderate physical activity in the past 7 days	1: Yes<br>2: No
**Moderate walk every day**	Whether the individual has walked for at least 10 minutes or more in the past 7 days	1: Yes<br>2: No
**Waist**	Manual measurement of waist circumference	Continuous
**Hip**	Manual measurement of hip circumference	Continuous
**Waist-Hip ratio**	Waist circumference/Hip circumference	Continuous
**Body Fat**	Automatic measurement, body fat percentage measurement value, with the unit of measurement being %	Continuous
**Visceral fat**	Automatic measurement, visceral fat percentage measurement value, with the unit of measurement being %	Continuous

**Pulse**	Automatic measurement, the measured value of cardiac pulsation frequency, with the unit of measurement being times per minute	Continuous
**BMI**	Automatic measurement, BMI	Continuous
**Systolic blood pressure**	Automatic measurement, individual pulse pressure measurement value, mmHg, takes the average of three times	Continuous
**Diastolic blood pressure**	Automatic measurement, individual pulse pressure measurement value, mmHg, takes the average of three times	Continuous

Table S3. Incident events and incidence rate of five common cancers in FuSion discovery cohort

	Incident events	Crude IR#			ASIR (China 2000)		
		Overall	Male	Female	Overall	Male	Female
**Full discovery cohort**							
Lung cancer	167	124.64	189.70	86.46	33.30	44.42	21.47
Esophageal cancer	132	98.57	163.71	60.40	25.95	35.07	16.26
Gastric cancer	137	102.31	192.09	49.73	29.95	40.33	18.92
Liver cancer	74	55.16	88.69	35.49	14.05	20.20	7.51
Colorectal cancer	85	63.41	113.02	34.32	18.27	25.73	10.33
**Training set**							
Lung cancer	117	124.33	168.51	98.15	35.14	43.57	26.19
Esophageal cancer	90	95.68	166.00	54.12	25.77	35.18	15.77
Gastric cancer	92	97.81	174.55	52.44	31.92	39.87	23.47
Liver cancer	55	58.38	99.90	33.79	15.27	22.42	7.68
Colorectal cancer	56	56.10	96.97	31.90	15.91	24.43	6.86
**Test set**							
Lung cancer	50	125.37	240.75	59.19	29.06	46.63	10.38
Esophageal cancer	42	105.38	158.21	75.04	26.60	35.12	17.54
Gastric cancer	45	112.93	234.33	43.41	25.74	41.93	8.53
Liver cancer	19	47.57	61.73	39.43	11.37	15.30	7.20
Colorectal cancer	29	68.56	129.58	33.50	20.17	23.14	17.00

IR: Incidence Rate;ASIR: Age Standardized Incidence Rate; Reference Panel: 2000 population census of China.

#per 100,000 person years.

All incidents were diagnosed before 2021-12-31

Table S4. Summary of 49 biomarkers in discovery and validation cohort

Variables	Discovery cohort			Validation cohort
	**Overall**	**Training set**	**Test set**	**Validation set**
	N = 16138	N = 11296	N = 4842	N = 26058
**A/G (mean (SD))**	2.03 (0.49)	2.04 (0.47)	2.03 (0.54)	1.93 (0.45)
**CRP (mean (SD))**	1.56 (3.91)	1.56 (3.83)	1.56 (4.11)	1.84 (4.58)
**GGT (mean (SD))**	25.65 (29.83)	25.73 (29.62)	25.45 (30.31)	29.07 (33.75)
**ALT (mean (SD))**	16.97 (15.22)	17.05 (16.26)	16.80 (12.46)	24.88 (19.04)
**CO2 (mean (SD))**	21.29 (2.29)	21.31 (2.30)	21.25 (2.28)	22.85 (2.27)
**LDL (mean (SD))**	3.14 (0.88)	3.15 (0.88)	3.13 (0.87)	3.26 (0.89)
**HCY (mean (SD))**	12.18 (5.31)	12.21 (5.44)	12.11 (4.99)	13.39 (6.15)
**UREA (mean (SD))**	5.83 (1.65)	5.82 (1.64)	5.83 (1.67)	6.10 (1.62)
**URAC (mean (SD))**	291.63 (78.91)	292.07 (79.17)	290.59 (78.28)	308.10 (79.02)
**TBIL (mean (SD))**	12.70 (5.34)	12.73 (5.42)	12.65 (5.14)	16.07 (6.44)
**CL (mean (SD))**	105.89 (2.73)	105.88 (2.74)	105.93 (2.70)	104.89 (2.59)
**AMY (mean (SD))**	65.83 (23.18)	65.81 (23.44)	65.87 (22.55)	66.22 (23.81)
**GLOB (mean (SD))**	23.68 (4.05)	23.67 (4.08)	23.72 (3.97)	25.65 (4.37)
**TG (mean (SD))**	1.50 (1.02)	1.50 (1.03)	1.50 (1.02)	1.58 (0.95)
**ALB (mean (SD))**	46.58 (2.84)	46.60 (2.86)	46.53 (2.77)	47.64 (2.46)
**ALP (mean (SD))**	87.25 (26.09)	87.08 (25.76)	87.64 (26.84)	99.36 (29.52)
**P (mean (SD))**	1.23 (0.18)	1.23 (0.18)	1.23 (0.18)	1.20 (0.17)
**CREA (mean (SD))**	55.06 (23.59)	55.04 (23.37)	55.10 (24.09)	54.99 (15.94)
**EGFR (mean (SD))**	122.03 (23.77)	121.99 (23.62)	122.11 (24.12)	126.64 (26.62)
**CYS-C (mean (SD))**	0.74 (0.22)	0.74 (0.22)	0.74 (0.23)	0.78 (0.21)
**GLU (mean (SD))**	5.35 (1.35)	5.36 (1.38)	5.33 (1.27)	5.25 (1.48)
**Ca (mean (SD))**	2.37 (0.11)	2.37 (0.11)	2.37 (0.11)	2.43 (0.10)
**Na (mean (SD))**	144.57 (3.07)	144.57 (3.13)	144.57 (2.94)	144.93 (2.51)
**K (mean (SD))**	3.94 (0.35)	3.94 (0.35)	3.94 (0.35)	4.02 (0.35)
**Mg (mean (SD))**	0.84 (0.07)	0.84 (0.06)	0.84 (0.07)	0.88 (0.07)
**HDL (mean (SD))**	1.42 (0.33)	1.42 (0.33)	1.42 (0.33)	1.43 (0.33)
**T3 (mean (SD))**	1.67 (0.31)	1.67 (0.31)	1.68 (0.31)	1.63 (0.27)
**HCV-Ab (mean (SD))**	0.18 (0.63)	0.19 (0.69)	0.17 (0.44)	0.19 (0.85)
**HBsAg (mean (SD))**	20.08 (65.22)	20.07 (65.18)	20.10 (65.33)	16.44 (58.82)
**TSH (mean (SD))**	3.00 (3.61)	3.00 (3.79)	3.00 (3.15)	3.02 (3.76)
**FOL (mean (SD))**	12.87 (3.77)	12.89 (3.76)	12.83 (3.78)	12.72 (3.79)
**T4 (mean (SD))**	98.17 (18.30)	98.14 (18.23)	98.24 (18.47)	104.24 (18.59)
**FT3 (mean (SD))**	4.87 (0.90)	4.86 (0.88)	4.87 (0.94)	4.59 (0.72)
**FT4 (mean (SD))**	16.22 (3.03)	16.21 (2.97)	16.23 (3.16)	15.30 (2.86)
**AFP (mean (SD))**	2.95 (2.99)	2.94 (3.00)	2.95 (2.98)	3.24 (8.32)
**CEA (mean (SD))**	2.19 (2.80)	2.17 (1.47)	2.25 (4.59)	2.41 (2.71)

**NSE (mean (SD))**	10.99 (4.56)	10.99 (4.58)	10.97 (4.51)	11.52 (2.81)
**CA-125 (mean (SD))**	12.73 (10.72)	12.64 (10.71)	12.95 (10.75)	14.04 (26.35)
**CA-153 (mean (SD))**	8.98 (4.24)	8.96 (4.27)	9.01 (4.17)	9.73 (4.52)
**CA-199 (mean (SD))**	13.08 (12.78)	13.14 (13.77)	12.93 (10.13)	14.23 (15.53)
**CYFRA-211 (mean (SD))**	2.34 (1.01)	2.34 (1.01)	2.34 (1.01)	2.01 (0.89)
**ProGRP (mean (SD))**	40.29 (24.44)	40.13 (13.76)	40.67 (39.35)	45.07 (20.04)
**PGI (mean (SD))**	51.74 (24.88)	51.66 (24.81)	51.93 (25.04)	58.61 (28.34)
**PGI/II (mean (SD))**	4.51 (2.98)	4.53 (3.03)	4.48 (2.86)	5.13 (2.47)
**PGII (mean (SD))**	14.15 (9.65)	14.05 (9.50)	14.36 (9.99)	14.03 (10.54)
**INS (mean (SD))**	48.02 (32.37)	48.13 (33.90)	47.78 (28.48)	56.38 (35.93)
**FER (mean (SD))**	132.72 (118.92)	132.83 (119.16)	132.44 (118.38)	159.91 (137.09)
**SCC-Ag (mean (SD))**	0.93 (0.99)	0.93 (0.90)	0.92 (1.18)	0.82 (1.28)
**Anti-HP (%)**				
Negative	13449 (83.3)	9455 (83.7)	3994 (82.5)	21040 (80.7)
Weak positive	1177 (7.3)	798 (7.1)	379 (7.8)	2549 (9.8)
Positive	952 (5.9)	650 (5.8)	302 (6.2)	2047 (7.9)
Missingness	560 (3.5)	393 (3.5)	167 (3.4)	422 (1.6)

Table S5. Univariable Cox regression results for incident 5CAs

Characteristic	Hazard Ratio	95%CI	P-value
**Biomarkers**			
A/G	1	0.93-1.08	9.07E-01
CRP	1.06	1-1.12	6.15E-02
GGT	1.1	1.06-1.15	0
ALT	1.01	0.93-1.09	8.97E-01
CO2	1.11	1.02-1.2	1.34E-02
LDL	0.93	0.85-1.01	7.37E-02
HCY	1.14	1.09-1.2	2E-16
AST	1.07	1.04-1.11	0
UREA	1.09	1.02-1.16	6.40E-03
URAC	1.3	1.21-1.4	0
TCHO	0.96	0.89-1.04	3.64E-01
TBIL	1.13	1.07-1.2	1.00E-04
TP	0.81	0.74-0.87	0
CL	1.17	1.08-1.27	2.00E-04
AMY	1.07	1-1.14	6.40E-02
GLOB	0.92	0.85-1	4.30E-02
TG	0.98	0.9-1.07	6.64E-01
ALB	0.79	0.73-0.85	0
DBIL	1.11	1.07-1.15	0
ALP	1.12	1.04-1.21	1.90E-03
P	0.94	0.87-1.02	1.35E-01
CREA	1.06	1.04-1.09	0
EGFR	0.79	0.73-0.87	0
CYS-C	1.14	1.11-1.17	0
GLU	1.11	1.04-1.19	1.60E-03
Ca	0.92	0.85-1	3.88E-02
Na	1.2	1.11-1.3	0
K	0.94	0.86-1.02	1.13E-01
Mg	1.07	0.99-1.16	1.01E-01
IBIL	1.12	1.04-1.2	2.90E-03
HDL	1.04	0.96-1.12	3.59E-01
T3	0.99	0.91-1.07	7.44E-01
HCV-Ab	1.04	1-1.09	8.13E-02
HBsAg	1.15	1.08-1.22	0
TSH	1	0.93-1.09	9.06E-01
FOL	0.84	0.77-0.91	0
T4	1.01	0.94-1.1	7.24E-01

FT3	1.01	0.94-1.08	8.09E-01
FT4	1.03	0.96-1.11	3.77E-01
AFP	1.09	1.07-1.12	0
CEA	1.1	1.08-1.12	0
NSE	0.9	0.83-0.98	1.80E-02
CA-125	1	0.92-1.08	9.70E-01
CA-153	1.14	1.06-1.22	2.00E-04
CA-199	1.07	1.03-1.11	4.00E-04
CYFRA-211	1.32	1.26-1.39	0
ProGRP	1.06	1.02-1.1	3.70E-03
PG-I	1.11	1.04-1.2	4.10E-03
PGI/II	0.94	0.86-1.03	1.93E-01
PG-II	1.14	1.07-1.22	2.00E-04
INS	0.91	0.82-1.01	6.80E-02
FER	1.15	1.07-1.23	1.00E-04
SCC-Ag	1.05	1.01-1.09	1.11E-02
Anti-HP (Weak positive)	1.56	1.19-2.04	1.40E-03
Anti-HP(Positive)	1.34	0.97-1.84	7.60E-02
**Epidemiological Exposures**			
Age	1.1	1.09-1.11	0
Sex=Male	2.84	2.4-3.35	0
Marital status = Divorced/Widowed	1.56	1.18-2.06	1.90E-03
Marital status = Single	2.58	1.38-4.83	2.90E-03
Education status = Intermediate	0.94	0.6-1.5	8.08E-01
Education status = Basic	0.83	0.55-1.27	3.94E-01
Education status = Less than basic	0.83	0.53-1.3	4.18E-01
height	1.04	1.03-1.05	0
weight	1.01	1-1.02	3.50E-03
Tobacco smoking status = Ever	3.8	2.84-5.08	0
Tobacco smoking status = Smoker	3.12	2.62-3.72	0
Smoking pack-years	1.03	1.03-1.04	0
Use filter when smoking = Yes	0.59	0.47-0.74	0
Inhalation smoking = Throat	1.23	0.88-1.72	2.22E-01
Inhalation smoking = Lung	1.33	1.02-1.73	3.31E-02
Drinking status = Ever	3.35	2.31-4.87	0
Drinking status = Drinker	2.46	2.08-2.92	0
Tea consumption status = Yes	0.54	0.45-0.64	0
Heavy activity works = Yes	1.12	0.9-1.39	3.26E-01
Moderate activity works = Yes	0.72	0.61-0.86	2.00E-04
Moderate walk every day = Yes	1.06	0.89-1.25	5.32E-01

Waist	1.02	1.01-1.03	5.00E-04
Hip	0.99	0.98-1	1.14E-01
Body Fat	0.95	0.93-0.96	0
Visceral fat	1.05	1.04-1.07	0
Pulse	1	0.99-1	3.16E-01
BMI	0.97	0.95-1	3.38E-02
BMI category = Normal	0.88	0.45-1.71	7.00E-01
BMI category = Overweight	0.82	0.42-1.6	5.60E-01
BMI category = Obesity	0.64	0.32-1.29	2.10E-01
Waist-Hip ratio (per 0.1)	1.53	1.35-1.73	0
Systolic blood pressure	1.01	1-1.01	1.63E-02
Diastolic blood pressure	1	1-1.01	9.60E-02

Table S6. Variable definitions and transformation parameters for the PRIME model

Variable	Coefficient	Original Value Range	Input Type	Mean*	SD*
Age (per 5 years)	0.422	40–75	Original (raw)	–	–
Sex (1 = Male; 0 = Female)	0.558	0 or 1	Original (binary)	–	–
Smoking Pack-Years	0.014	0–150	Original (raw)	–	–
AFP (ng/mL)	0.053	0–200	Z-score transformed	2.979	2.340
CEA (ng/mL)	0.085	0–50	Z-score transformed	2.214	2.067
CYFRA-21-1 (ng/mL)	0.187	0–20	Z-score transformed	2.292	0.988
HBsAg (IU/L)	0.125	0–250	Z-score transformed	19.351	63.813

*: Mean and standard deviation (SD) values were derived from the original biomarker measurements in the training set and were used for Z-score transformation.

Table S7. Definition and distribution of new diagnosis cancer or precancerous lesion of different type of cancer.

Type of Diagnosis	High Risk	Intermediate Risk	Low Risk
**Lung cancer**			
Cancer (Screening)	7(0.27%)	3(0.06%)	0(0%)
Cancer (Medical system)	4(0.15%)	9(0.18%)	0(0%)
NSNs	40(1.52%)	49(1.00%)	7(0.51%)
**Esophageal cancer**			
Cancer (Screening)	16(1.11%)	3(0.08%)	0(0%)
Cancer (Medical system)	1(0.09%)	0(0%)	0(0%)
HGIN	3(0.26%)	3(0.12%)	0(0%)
Barrett's esophagus	4(0.34%)	5(0.21%)	1(0.12%)
**Gastric cancer**			
Cancer (Screening)	11(0.94%)	0(0%)	0(0%)
Cancer (Medical system)	3(0.26%)	2(0.08%)	0(0%)
HGIN	1(0.09%)	0(0%)	0(0%)
Gastric dysplasia	16(1.36%)	30(1.25%)	3(0.36%)
**Liver cancer**			
Cancer (Medical system)	3(0.07%)	2(0.04%)	0(0%)
Liver cirrhosis	5(0.18%)	11(0.22%)	1(0.07%)
**Colorectal cancer**			
Cancer (Screening)	9(0.78%)	9(0.38%)	1(0.12%)
Cancer (Medical system)	5(0.43%)	6(0.25%)	0(0%)
HGIN	0(0%)	1(0.04%)	0(0%)
Dysplasia	18(1.56%)	37(1.56%)	6(0.74%)

Table S8. Significant variables in 1:4 matched subgroup

Variable	Hazard Ratio	95%CI	P-value
CEA	1.08	1.06 - 1.1	6.03E-19
CYFRA-211	1.22	1.15 - 1.29	1.65E-10
AFP	1.06	1.04 - 1.09	3.33E-07
Smoking Pack-Years	1.01	1.01 - 1.02	6.99E-07
ALB	0.83	0.76 - 0.9	4.02E-06
CA-199	1.2	1.11 - 1.3	8.57E-06
CL	1.17	1.09 - 1.27	4.47E-05
HBsAg	1.14	1.07 - 1.21	7.63E-05
NSE	0.88	0.81 - 0.96	3.25E-03
Na	1.12	1.04 - 1.22	4.60E-03

Table S9. Predictive ability of PRIME after restricted population after enrollment

	Training set	Test set
**Dropped out first 2 years**	0.782 (95%CI:0.747-0.817)	0.724 (95%CI: 0.665-0.783)
**Dropped out first 3 years**	0.784 (95%CI:0.743-0.826)	0.726 (95%CI:0.654-0.797)

Table S10. Predictive ability of PRIME in each cancer types

	Training set	Test set
Lung cancer	0.819 (95% CI: 0.766-0.872)	0.815 (95% CI: 0.766-0.872)
Esophageal cancer	0.784 (95%CI:0.743-0.826)	0.726 (95%CI:0.654-0.797)
Gastric cancer	0.789 (95% CI: 0.732-0.845)	0.774 (95% CI: 0.732-0.845)
Liver cancer	0.870 (95% CI: 0.807-0.933)	0.818 (95% CI: 0.807-0.933)
Colorectal cancer	0.716 (95% CI: 0.610-0.822)	0.620 (95% CI: 0.610-0.822)

Table S11. Stratified analysis of PRIME by sex and age group

Category	AUROC (95%CI)
**Sex**	
Female	0.805 (95%CI:0.76-0.85)
Male	0.713 (95%CI:0.667-0.758)
**Age group**	
50-	0.87 (95%CI:0.816-0.924)
60-	0.732 (95%CI:0.671-0.793)
70-	0.679 (95%CI:0.621-0.737)
70+	0.732 (95%CI:0.549-0.915)

Table S12. Comparation of high-risk participant between follow-up attendance status

Variables	Overall	Attend follow-up	Not Attend follow-up	P-value
	N = 4200	N = 2863	N = 1337	
Age (mean (SD))	64.30 (4.22)	64.33 (4.08)	64.24 (4.50)	0.519
Sex = Male	3705 (88.21)	2574 (89.90)	1131(84.59)	<0.001
Smoking Pack-Years (mean (SD))	21.75 (21.06)	22.27 (20.99)	20.64 (21.17)	0.02
AFP (mean (SD))	0.20 (2.00)	0.14 (1.12)	0.33 (3.14)	0.004
HBsAg (mean (SD))	0.13 (1.19)	0.12 (1.17)	0.17 (1.24)	0.223
CYFRA-211 (mean (SD))	0.61 (1.39)	0.59 (1.40)	0.65 (1.37)	0.255
CEA (mean (SD))	0.42 (1.75)	0.38 (0.96)	0.50 (2.76)	0.045

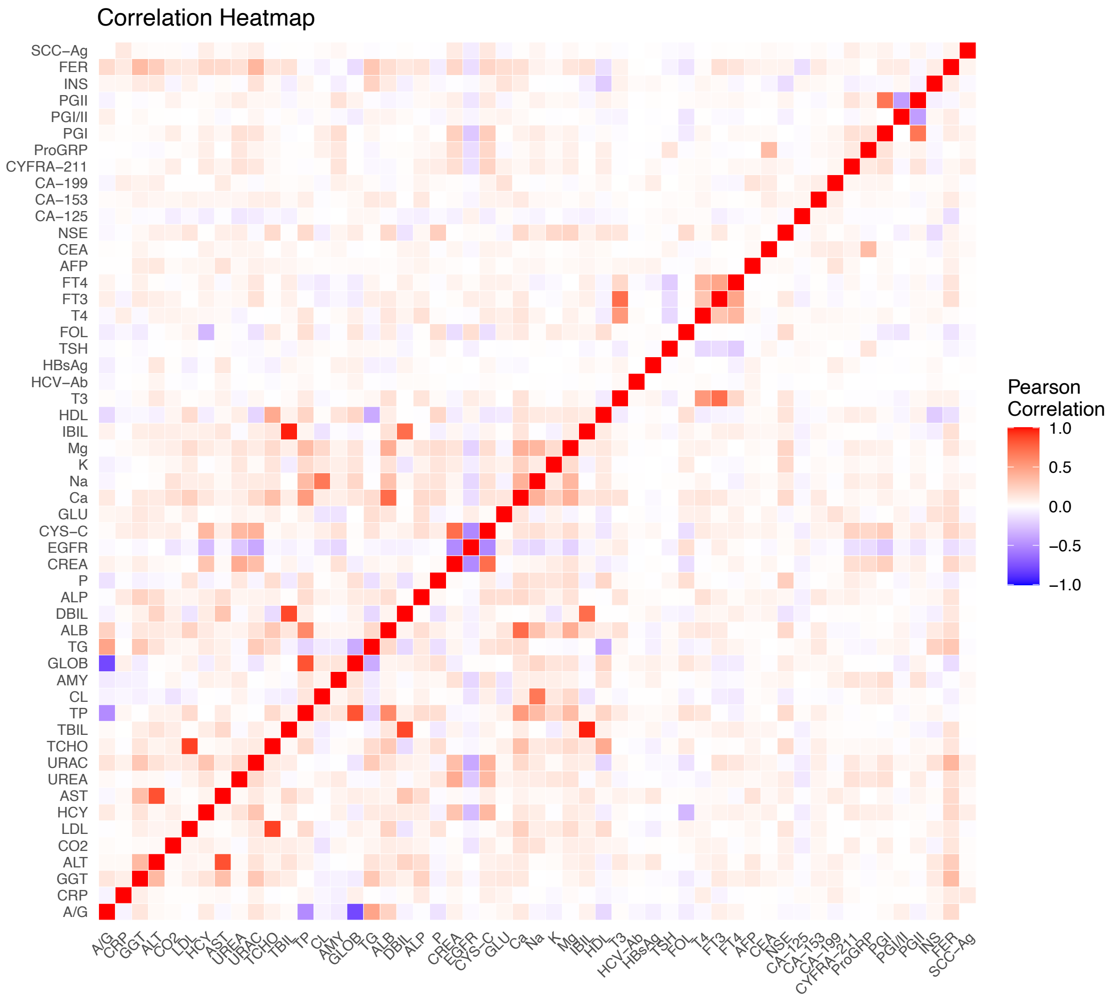

Figure S1. Correlation heatmap among 54 biomarkers.

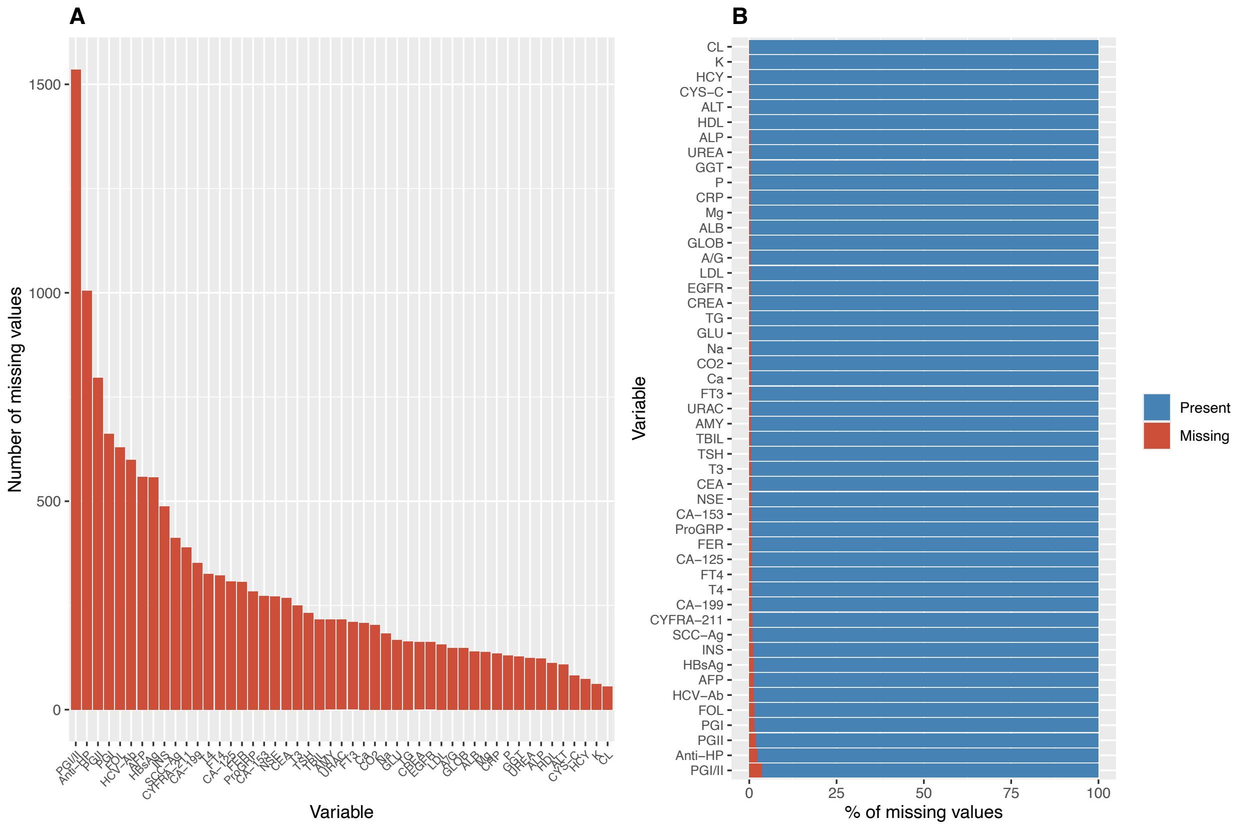

Figure S2. Missing counts (percentage, exposure name) of 49 biomarkers. (A) Missing Counts. (B) Missing percentage.

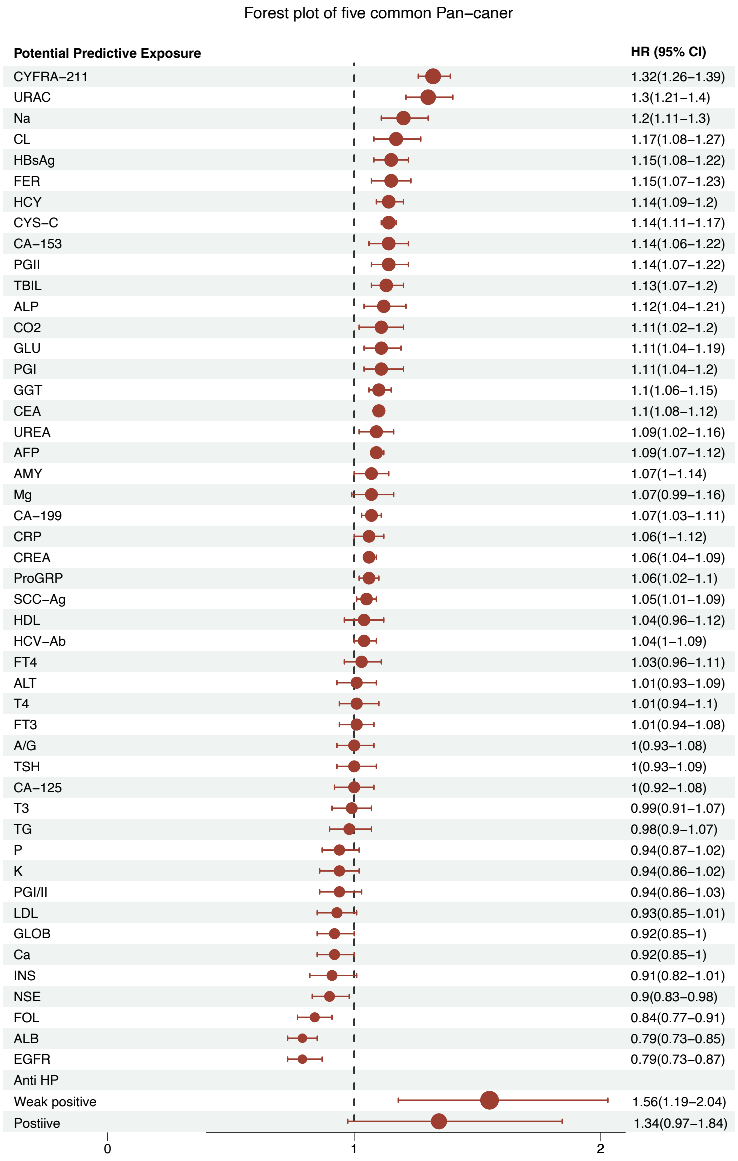

Figure S3. Forest plot of all 49 biomarkers with cancer incident risk.

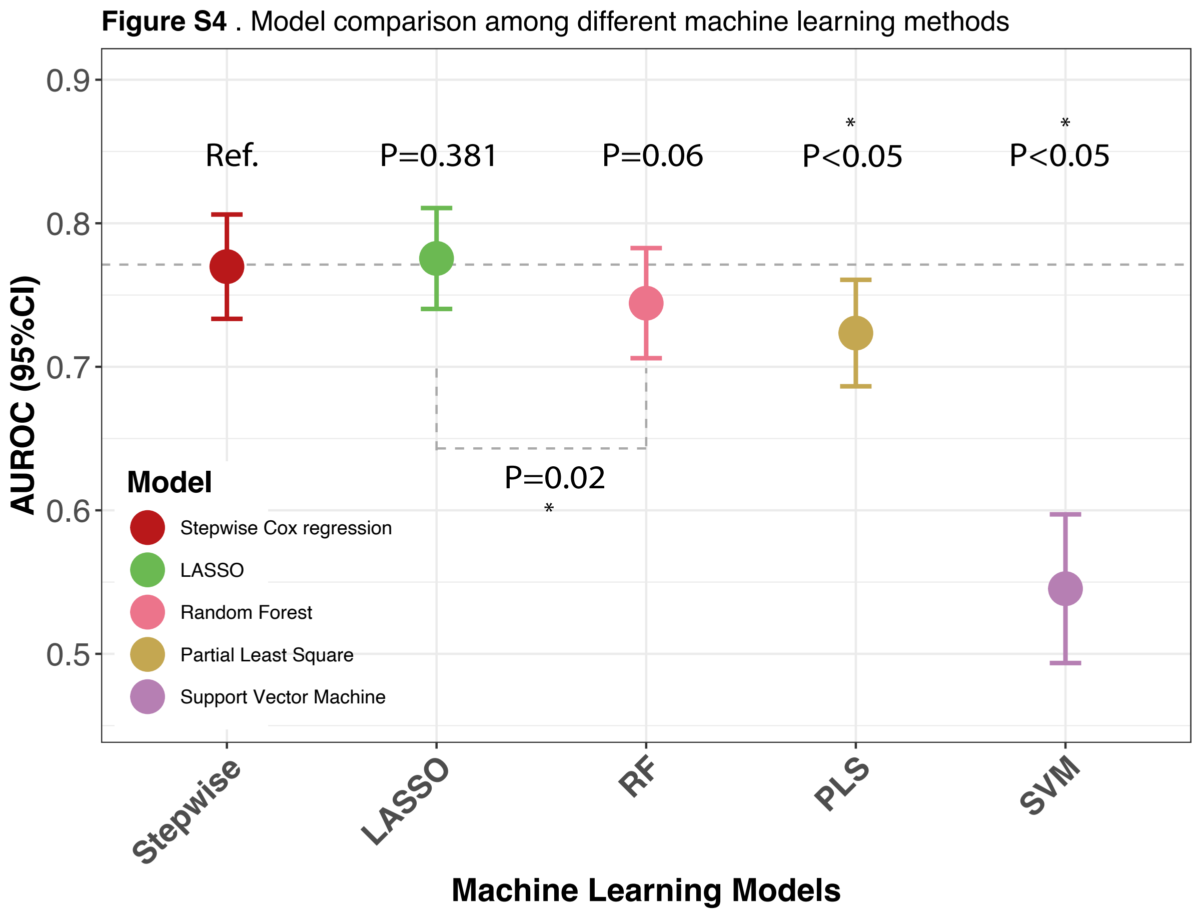

Figure S4. Model Comparison among five different machine learning procedures. (LASSO).

*: Statistical significant.

A

B

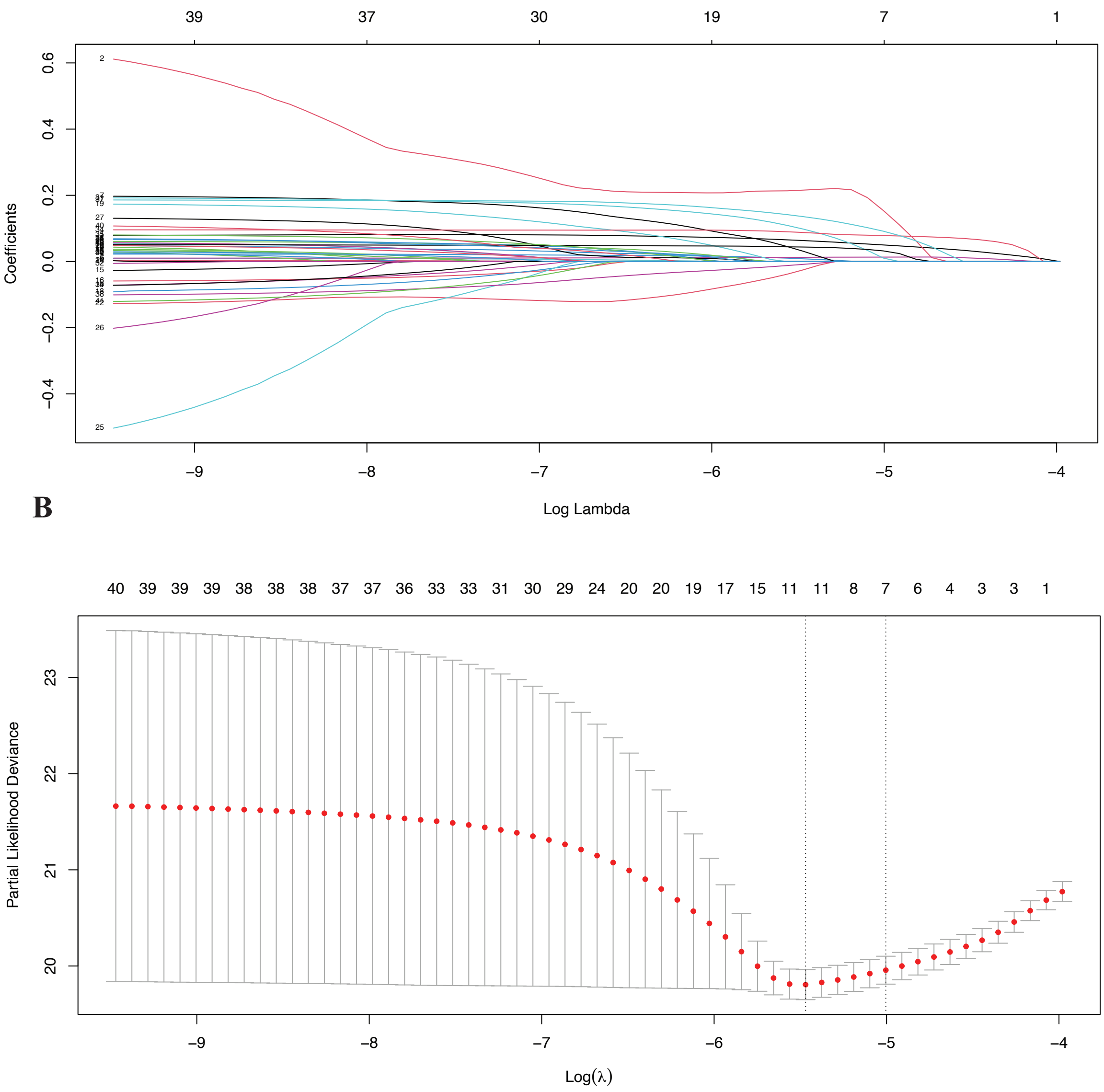

Figure S5. Variable Selection via L1 Absolute Shrinkage and Selection Operator (LASSO).

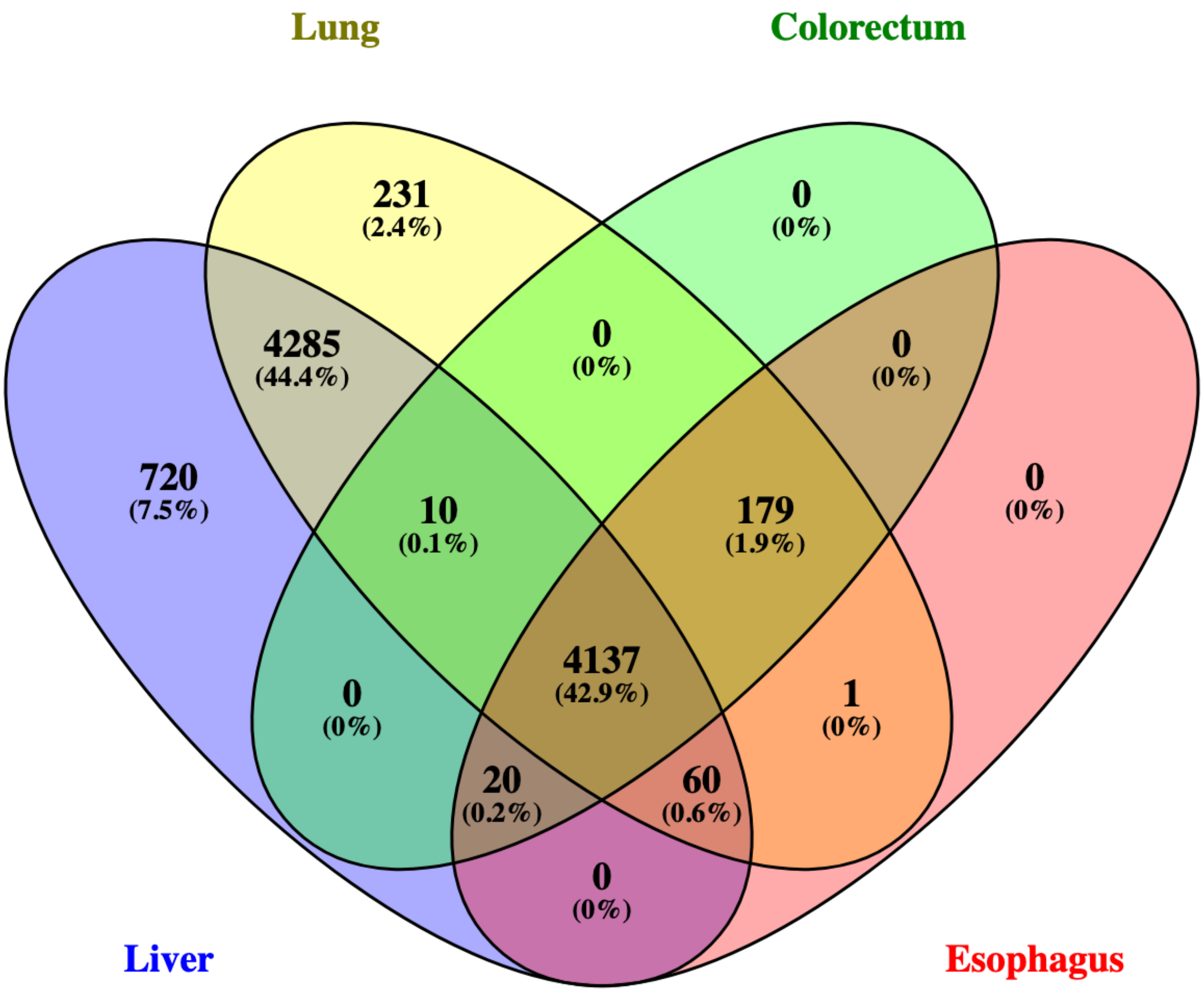

Figure S6. The Venn chart of people participating in medical examinations.

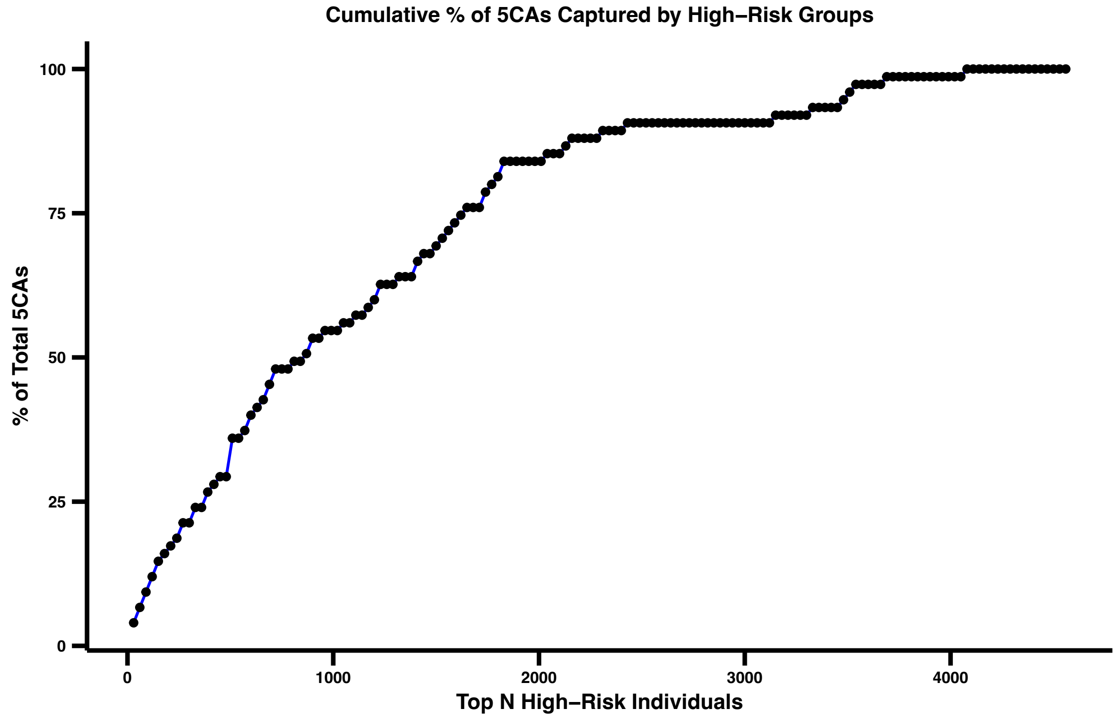

Figure S7. Screening curve between participants needed to screen and percentage of cancer incidents captured.
